# Rethinking International University Ranking Systems in the Context of Academic Public Health

**DOI:** 10.3389/ijph.2022.1605252

**Published:** 2022-08-29

**Authors:** Adeline Dugerdil, Lara Sponagel, Awa Babington-Ashaye, Antoine Flahault

**Affiliations:** Institute of Global Health, Faculty of Medicine, University of Geneva, Geneva, Switzerland

**Keywords:** public health, university students, ranking methodology, public health challenges, academic performance

The first international university ranking system created in 2003, The Shanghai Academic Ranking of World Universities, marked the systematic development of international university ranking systems. Up until today, the rationale behind their existence represents a crucial question. According to Marginson [1], quoting Adam Smith: “The desire of bettering our condition… comes with us from the womb, and never leaves us till we go into the grave.” Indeed, the majority of human beings strive for improvement and in doing so compare themselves with their fellow peers. Who says comparison, says ranking systems.

The primary aim of rankings was to provide an objective, transparent and easily understandable tool to promote a virtuous competition between the universities and to enable them to reach excellence. Due to the globalization of the academia, ranking systems have become essential for students, in order to choose the most suitable institution for their education, but also for universities to recruit their academic staff. As highlighted by Dill et al. [2], quoting the White Paper on higher education in the United Kingdom: “Market competition could be an important driver of academic quality, if appropriate university information can be provided to help inform student choice.” Moreover, rankings can represent a “promotion tool” for universities [3]. Apart from these honorable aims, university ranking systems have been subject to an extensive amount of criticism.

In fact, financial and reputational reasons may be a factor for sustaining such ranking systems. This sensitive issue was underlined by Butler [4], who quoted the Times Higher Education World University Rankings editor Ann Mroz declaring: “We are very much aware that national policy and multimillion-pound decisions are influenced by these rankings.” Marmolejo [5] also highlighted that “Nowadays, it is common to observe entire policies and programs from governments apparently more concerned with the position in the rankings than on the relevance of their tertiary education institutions.” According to ranking experts, these ranking systems are not always representative of the quality of the institutions because of major biases. Gadd [6] explained that “Rankings apply a combination of indicators that might not represent universities’ particular missions, and often overlook societal impact or teaching quality.” This overview of experts’ opinions shows that rankings have been diverted into a tool to establish the reputation of schools and for accumulating funding.

Regarding public health and according to the World Health Organization (WHO) [7], there are at least six major challenges to be addressed by in the 21st century: “Economic crisis; Widening inequalities; Ageing population; Increasing levels of chronic disease; Migration and urbanization; Environmental damage and climate change.” This highlights the importance of public health, its responsibilities to society and the wide range of domains that need to be covered by university education in this field. The university course in public health is thus distinguished in several ways. First of all, public health is a discipline that includes many others, as for example epidemiology or infectious diseases, pointing out its very broad field. Secondly, public health stands out for its societal involvement, for example through the COVID-19 pandemic when public health experts have regularly found themselves as close advisors to political decision-makers. Thirdly, public health is an inter- and transnational discipline, which is easily understandable when one looks at the WHO challenges previously mentioned. Therefore, in order to promote the development of public health as a leading discipline, it seems clear that the development of a specific ranking for medical and health sciences is a mandatory.

In this context, a scoping review on international university ranking systems was conducted by a team of public health researchers [8]. The study’s research question was: Are international university ranking systems focused on and adapted to the disciplines of medical and health sciences? The results highlighted that an international university ranking system specifically designed for medical and health sciences does not exist, even if some global rankings, developed initially to evaluate an institution as a whole, try to adapt their indicators to certain disciplines. This research also underlined the many gaps that exist in ranking systems, for example their methodological challenges.

As already pointed out by many authors, shortcomings of ranking systems make them less reliable than one might initially think. The purpose of this commentary is certainly not to provide an exhaustive list of these but rather to draw attention to some of the most striking limits. Firstly, many ranking systems are highly dependent on reputational surveys and thus cannot claim to represent an objective and reproducible tool to provide fair information to all stakeholders. Secondly, ranking systems are quite subjective and biased in many ways, whether by the choice of indicators and their weighting (emphasized by Stoupas et al. [9]) or by the lack of inclusion of certain indicators. Thirdly, ranking systems are not very inclusive in regards to low- and middle-income countries. Among the many reasons why this is the case, the majority of rankings are solely based on English-speaking databases and restrict the use of other languages (as highlighted by Selten et al. [10]). These previous considerations have led to the following bitter conclusion: rankings are often biased, misused and linked (more than one would like to believe) to a real economic system. Nevertheless, the fact that most ranking specialists are aware of the existing challenges highlights the willingness to improve the situation. Several teams have already proposed promising alternatives, for example by creating a ranking where universities themselves could choose the weighting of the indicators [11].

Following this brief review of ranking systems, the question raised is the following: what if the fundamental problem with ranking systems’ shortcomings lies in the fact that these rankings are not specifically dedicated to one discipline? Indeed, the aforementioned scoping review showed that ranking systems were initially built with the idea of classifying institutions as one entity. These ranking systems were then adapted (more or less well) to different disciplines. What if by pursuing the goal to be essentially globalized, ranking systems were to miss the very essence of the disciplines and thus compare the incomparable? Regarding different disciplines, and especially public health, it might be beneficial to think globally in terms of defining the menu of possible indicators but also to specify the terms in which each ranking applies. It is only by accepting to play this balancing act between global thinking and specific adaptation to each discipline that robust and quality ranking systems could be developed and be able to endure over time.

## References

[1] Marginson S. University Rankings and Social Science: University Rankings and Social Science. Eur J Educ (2014) 49:45–59. 10.1111/ejed.12061

[2] Dill DD Soo M. Academic Quality, League Tables, and Public Policy: A Cross-National Analysis of University Ranking Systems. High Educ (2005) 49:495–533. 10.1007/s10734-004-1746-8

[3] Aguillo IF Bar-Ilan J Levene M Ortega JL. Comparing university Rankings. Scientometrics (2010) 85:243–56. 10.1007/s11192-010-0190-z

[4] Butler D. University Rankings Smarten up. Nature (2010) 464:16–7. 10.1038/464016a 20203575

[5] Marmolejo F. Are We Obsessed with university Rankings? (2022). Available from: https://blogs.worldbank.org/education/are-we-obsessed-university-rankings (accessed Jun 7, 2022).

[6] Gadd E. University Rankings Need a Rethink. Nature (2020) 587:523. 10.1038/d41586-020-03312-2 33235367

[7] World Health Organization (WHO). Public Health Services (2022). Available from: https://www.euro.who.int/en/health-topics/Health-systems/public-health-services (accessed May 9, 2022).

[8] Dugerdil A Sponagel L Babington-Ashaye A. International University Ranking Systems and Their Relevance for the Medical and Health Sciences - A Scoping Review. Int J High Educ (2022). 10.3389/ijph.2022.1605252 PMC9464806 36105177

[9] Stoupas G Sidiropoulos A Katsaros D Manolopoulos Y. When Universities Rise (Rank) High into the Skyline. COLLNET J Scientometrics Inf Management (2021) 15:241–58. 10.1080/09737766.2021.1955419

[10] Selten F Neylon C Huang C-K Groth P. A Longitudinal Analysis of university Rankings. Quant Sci Stud (2020) 1:1109–35. 10.1162/qss_a_00052

[11] Kudela J. Mixed-Integer Programming Model for Ranking Universities: Letting Universities Choose the Weights. MENDEL (2021) 27:41–8. 10.13164/mendel.2021.1.041

